# Lippincott’s Magazine

**Published:** 1874-03

**Authors:** 


LIPPINCOTT'S MAGAZINE.—The April number of this favorite monthly, finely illustrated and overflowing with literary excellencies, is before us. Lippincott is always entertaining, always comes up to the expectations of the reader, and is one the purest and best of our literary magazine. Price $4.00 per annum. J. B. Lippincott & Co., Philadelphia.

